# Prostate cancer: Value of the PI-FAB score and volume-adjusted PSA density in recurrence assessment after HIFU

**DOI:** 10.1016/j.ejro.2026.100787

**Published:** 2026-06-25

**Authors:** Clara Elsner, Antonia M. Pausch, Toni Rabadi, Tabea Borde, Niels J. Rupp, Daniel Eberli, Ashkan Mortezavi, Priska Heinz, Ulrike Held, Andreas M. Hötker

**Affiliations:** a Diagnostic and Interventional Radiology, University Hospital Zurich, Rämistrasse 100, Zurich 8091, Switzerland; b Department of Pathology and Molecular Pathology, University Hospital Zurich, Schmelzbergstrasse 12, Zurich 8091, Switzerland; c Faculty of Medicine, University of Zurich, Rämistrasse 71, Zurich 8006, Switzerland; d Department of Urology, University Hospital Zurich, Frauenklinikstrasse 10, Zurich 8091, Switzerland; e Epidemiology, Biostatistics and Prevention Institute (EBPI), Department of Biostatistics, University of Zurich, Hirschengraben 84, Zurich 8001, Switzerland

**Keywords:** HIFU, VaPSA-D, PI-FAB, Prostate cancer, MpMRI

## Abstract

**Purpose:**

To evaluate the diagnostic performance of the Prostate Imaging after Focal Ablation (PI-FAB) score on multiparametric MRI in combination with volume-adjusted PSA density (vaPSA-D, derived from volumetrically assessed residual vital prostate tissue) for detecting in-field recurrence after HIFU ablation of localized prostate cancer.

**Methods:**

In this retrospective single-center study, 117 men treated with HIFU underwent follow-up mpMRI and prostate biopsies at 6 (n = 99), 12 (n = 74), and 36 (n = 52) months. PI-FAB scores and vaPSA-D were assessed independently. Diagnostic performance was evaluated using biopsy-based histopathology as the reference standard.

**Results:**

PI-FAB demonstrated good diagnostic performance, particularly at 36 months (AUC 0.92). vaPSA-D showed increasing accuracy over time (AUC 0.64, 0.82, 0.84 at 6, 12, and 36 months; overall 0.78), outperforming PI-FAB at 12 months. Integrating vaPSA-D into PI-FAB subgroups significantly enhanced recurrence assessment: PI-FAB 1: sensitivity/specificity 75%/79% (threshold 0.14 ng/ml); PI-FAB ≥ 2: sensitivity/specificity 65%/85% (0.18 ng/ml); PI-FAB 3: sensitivity/specificity 67%/90% (0.18 ng/ml).

**Conclusion:**

Both PI-FAB and vaPSA-D provide reliable recurrence assessment after HIFU, with complementary strengths: PI-FAB performs best at later follow-up stages, whereas vaPSA-D maintains high sensitivity throughout. Their combined application offers the most accurate evaluation of post-HIFU recurrence and may help reduce unnecessary biopsies.

## 1 Introduction

Over the last decade, high-intensity focused ultrasound (HIFU) focal therapy has emerged as an alternative treatment option for patients with localized prostate cancer, with the aim of providing oncological control while preserving erectile and urinary function [1]. It is a minimally invasive procedure designed to selectively destroy a localized area of prostate cancer while preserving the surrounding non-cancerous tissue with promising oncological and functional outcomes [2], [3]. Due to the relative novelty of HIFU, follow-up protocols to monitor treatment success lack standardization and are still subject of ongoing studies [4], [5]. Conventional postinterventional monitoring using prostate-specific antigen (PSA) levels is challenging due to individually varying amounts of residual viable prostate tissue as well as inflammation after focal treatment and should primarily be used for longitudinal trend assessment [5]. Multiparametric MRI (mpMRI) of the prostate may aid in post-treatment monitoring, as Giganti et al. recently introduced the Prostate Imaging after Focal Ablation (PI-FAB) score - the first standardized system for evaluating the prostate after focal ablation therapy [6]. This system is based on the assessment of key imaging features that indicate residual or recurrent prostate cancer within/adjacent to the treated area and has been validated in several studies [6], [7], [8], [9], [10]. However, challenges remain as the authors note that a PI-FAB score of 2 represents an equivocal category that requires clinical assessment and PSA monitoring to determine the need for biopsy [6].

In the pre-biopsy setting, the MRI-derived PSA density (PSA-D) is commonly used to guide biopsy decision in patients with elevated PSA and indeterminate MRI findings. It is calculated using the “ellipsoid formula” to estimate prostate volume, based on T2-weighted images [11], [12], [13]. However, this formula is not applicable in cases where the prostate has undergone significant structural changes, such as those induced by HIFU therapy - including edema, necrosis, and in later stages fibrosis and retraction of the prostatic capsule [14]. Still, MRI in the post-treatment setting allows for volumetry of the amount of remaining vital prostate tissue and therefore the calculation of a volume-adjusted PSA density (vaPSA-D).

Therefore, the objective of our study was (1) to assess the diagnostic accuracy of the PI-FAB score and volume-adjusted post-treatment PSA-D to detect in-field cancer recurrence in a cohort of patients who underwent HIFU therapy and then (2) to assess the ability of vaPSA-D to further refine the PI-FAB score, allowing for more precise detection or exclusion of in-field cancer recurrence and improved individual clinical management.

## 2 Methods

### 2.1 Study design and reference standard

This IRB-approved retrospective single-center study included patients ≥ 18 years, who underwent high-intensity focused ultrasound (HIFU) therapy for localized prostate cancer (PCa) at our institution between May 2014 and October 2024 for a low- to intermediate-risk non-metastatic PCa defined by an ISUP/WHO grade group of ≤ 3, had provided a valid general consent and had available histopathological data from prostate biopsy.

A subset of patients included in the present study was derived from a prospective intervention trial on focal HIFU therapy for the treatment of prostate cancer (ClinicalTrials.gov identifier NCT02265159). 83 of the 117 patients in the current cohort have therefore been included in prior publications by Pausch et al., Kaufmann et al., Burger et al., Mortezavi et al., and Spitznagel et al. [3], [7], [8], [15], [16], [17].

While active surveillance was the preferred recommendation for patients with ISUP/WHO grade group 1 disease, some patients opted for HIFU therapy based on personal preference.

Preprocedural baseline mpMRIs were available for every patient. Follow-up mpMRIs were conducted at 6, 12, and 36 months post-HIFU treatment. Saturation biopsies along with targeted biopsies of the ablation zone were performed in a standardized manner at the same time points, and all histopathological results were obtained, which served as standard of reference. All prostate biopsies were carried out by board-certified urologists and reviewed by specialized genitourinary pathologists. A clinically significant in-field cancer recurrence was defined as a lesion located within or directly adjacent to the ablation zone with a Gleason score of ≥ 3 + 4 (ISUP/WHO grade group ≥ 2 [18]). Out-of-field clinically significant prostate cancer (csPCa) was recorded but considered outside the scope of this study.

Patients who declined MRI or biopsy were excluded from analyses at the respective follow-up time point. The study flowchart is provided in Supplementary Figure S1. A total of 99, 74, and 52 patients completed the 6-, 12-, and 36-month follow-up examinations, respectively. Baseline clinical data, including patient age and PSA levels, were collected from clinical and radiology information systems.

### 2.2 Multiparametric prostate MRI acquisition and analysis

All multiparametric MRIs (mpMRIs) were conducted using 3 T MR scanners (MAGNETOM Skyra or Vida fit, Siemens Healthineers, Erlangen, Germany). The imaging protocol adhered to the current Prostate Imaging Reporting and Data System (PI-RADS) guidelines [19] at the time of acquisition, with occasional use of an endorectal coil.

The mpMRI protocol included high-resolution T2-weighted (T2w) turbo spin echo (TSE) sequences in three planes, diffusion-weighted imaging (DWI) with b-values of 100, 600, and 1000 s/mm², a calculated b-value of 1400 s/mm², the apparent diffusion coefficient (ADC) map, and dynamic contrast-enhanced (DCE) MRI using gadoterate meglumine (Dotarem, Guerbet, Villepinte, France) as the contrast agent, administered at a dose of 0.1 mmol/kg body weight.

All MRI examinations were deemed to be of sufficient diagnostic quality; no cases were excluded due to suboptimal image quality.

A radiologist with three years of experience (blinded for review) in reading prostate MRIs (250 prostate MRIs annually) reviewed the mpMRIs using Mint Lesion™ software (Mint Medical GmbH, Heidelberg, Germany) and manually performed slice-by-slice volumetric segmentation of the residual viable prostate tissue post-HIFU using T2-weighted sequences (T2w) in the transverse plane, with dynamic contrast-enhanced sequences (DCE) employed as needed for additional reference.

Another radiologist (blinded for review) with five years of experience in reading prostate MRIs (300 prostate MRIs annually), assessed the mpMRIs on a PACS workstation to assign a PI-FAB score [13] to each MRI. This radiologist was blinded to clinical and histopathological data, only being informed that the imaging was conducted as part of HIFU follow-up. Preprocedural baseline and previous post-HIFU mpMRIs were available for comparison purposes.

The PI-FAB scoring system, described in detail by Giganti et al., uses a 3-point scale based on an analysis of DCE, DWI/high b-value sequences and the ADC map, and T2w images, with a PI-FAB score of 3 indicating a high likelihood of residual or recurrent prostate cancer [6].

### 2.3 Statistical analysis

Descriptive statistics included median and interquartile range for continuous variables, as well as number and percentage of total for categorical variables.

Dichotomized PI-FAB scores were defined as follows: A PI-FAB score of ≥ 2 was classified as "test positive", while a PI-FAB score of 1 was classified as "test negative".

The diagnostic performance of the dichotomized PI-FAB score for detecting in-field recurrences on follow-up mpMRI scans at 6, 12, and 36 months post-HIFU was analyzed on a per-patient level. Subgroup analyses based on PI-FAB categories (PI-FAB 1, PI-FAB ≥ 2, and PI-FAB 3) were subsequently performed to assess the added diagnostic value of vaPSA-D within different clinical risk strata.

To assess diagnostic accuracy of PI-FAB scores, the histopathologic biopsy results served as reference standard. Sensitivity, specificity, positive predictive value (PPV), and negative predictive value (NPV) were calculated along with their 95% confidence intervals (CI).

Multiple observations in the same patients were evaluated over time. To account for correlation in the longitudinal data, sensitivity and specificity were additionally estimated using generalized estimating equations (GEE) with exchangeable correlation structure. Different GEE models were compared based on the quasi-likelihood information criterion (QIC). Models with smaller QIC values are preferred.

The Youden index was used to derive a threshold in predicted probability for the outcome in-field cancer recurrence.

All statistical analyses were performed using the software R (version 4.5.1 [20]) in combination with dynamic reporting using R Markdown in a fully scripted analysis for reproducibility. All analyses were pre-specified in a statistical analysis plan before data base closure.

## 3 Results

### 3.1 Patient characteristics

The median patient age at time of baseline MRI was 66 years (IQR 61 – 70) with a median PSA value of 6.07 ng/ml (IQR 4.77 – 7.82). Prior to HIFU treatment, the distribution of ISUP/WHO Gleason grade groups was as follows: grade group 1 in 5/117 patients (4.3%), grade group 2 in 77/117 patients (65.8%) and grade group 3 in 35/117 patients (29.9%). A summary of patient characteristics before HIFU treatment is presented in Table 1.

**Table 1:** Overview of patient demographics., PSA: prostate specific antigen, PI-RADS: Prostate Imaging Reporting and Data System, HIFU: high-intensity focused ultrasound, ISUP/WHO: International Society of Urological Pathology/World Health Organization.

*Patient Demographics (n = 119)*	
Age (years), median (IQR)	66 (61 – 70)
Pre-HIFU PSA (ng/ml), median (IQR)	6.07 (4.77 – 7.82)
Pre-HIFU highest PI-RADS score, n (%)	
PI-RADS 2	8 (6.8)
PI-RADS 3	12 (10.3)
PI-RADS 4	62 (53.0)
PI-RADS 5	35 (29.9)
Pre-HIFU highest ISUP/WHO grade, n (%)	
ISUP/WHO grade 1 (Gleason 3 + 3)	5 (4.3)
ISUP/WHO grade 2 (Gleason 3 + 4)	77 (65.8)
ISUP/WHO grade 3 (Gleason 4 + 3)	35 (29.9)

At 6 months after HIFU, 99 patients underwent follow-up mpMRI with subsequent biopsy, revealing in-field residual or recurrent csPCa in 7 of 99 men (7.1%). At the 12-month follow-up, mpMRI with biopsy was performed in 74 men with in-field residual or recurrent disease in 15 of 74 (20.3%) men. At 36 months follow-up, mpMRI and biopsy were available for 52 men, with 7 of 52 (13.5%) showing in-field recurrent disease (Table S1 in the supplements).

#### 3.1.1 Diagnostic performance of PI-FAB score

The PI-FAB score with a cut-off at ≥ 2 for test positive, demonstrated sensitivities of 43%, 47% and 100% and specificities of 96%, 97%, and 84% at 6, 12 and 36 months (NPV: 96%, 88%, 100%; PPV: 43%, 78%, 50%, respectively) in detection of residual or recurrent prostate cancer, resulting in an overall accuracy of 92%, 86% and 87%. AUC values were 0.70, 0.72 and 0.92 at 6, 12 and 36 months after HIFU (see Table 2). When adjusting for multiple observations in the same patient, an overall sensitivity of 57%, specificity of 93% and AUC value of 0.76 was found.

**Table 2:** Statistical metrics evaluating the diagnostic performance of the PI-FAB score at different follow-up intervals (6, 12 and 36 months) post-HIFU treatment. PI-FAB scores from follow-up MRI scans were compared with histopathologic findings of saturation and targeted prostate biopsies, which served as the reference standard. HIFU: high-intensity focused ultrasound. m: months. SENS: sensitivity. SPEC: specificity. PPV: positive predictive value. NPV: negative predictive value. ACC: accuracy. AUC: area under the curve. 95% CI: 95% confidence interval.

*Diagnostic performance of the PI-FAB score*						
Follow-up interval post-HIFU (n)	**SENS** **(95% CI)**	**SPEC (95% CI)**	**PPV** **(95% CI)**	**NPV** **(95% CI)**	**ACC** **(95% CI)**	**AUC** **(95% CI)**
6 m (n = 99)						
	0.43 (0.10, 0.82)	0.96 (0.89, 0.99)	0.43 (0.10, 0.82)	0.96 (0.89, 0.99)	0.92 (0.85, 0.96)	0.70 (0.53, 0.91)
12 m (n = 74)						
	0.47 (0.21, 0.73)	0.97 (0.88, 1.00)	0.78 (0.40, 0.97)	0.88 (0.77, 0.95)	0.86 (0.77, 0.93)	0.72 (0.59, 0.83)
36 m (n = 52						
	1.00 (0.59, 1.00)	0.84 (0.71, 0.94)	0.50 (0.23, 0.77)	1.00 (0.91, 1.00)	0.87 (0.74, 0.94)	0.92 (0.87, 0.98)

#### 3.1.2 Diagnostic performance of vaPSA-D

With a threshold of 0.11, 0.13 and 0.15 ng/ml at 6, 12 and 36 months after HIFU, the vaPSA-D showed a sensitivity of 71%, 87%, 100% and correspondingly an NPV of 96%, 96%, 100% (see Table 3 for full performance metrics).

**Table 3:** Statistical metrics evaluating the diagnostic performance of the MRI-based vaPSA-D at different follow-up intervals (6, 12 and 36 months) post-HIFU treatment. For each time point, an individual optimal cut-off value was calculated with maximized Youden’s Index. vaPSA-D from follow-up MRI scans was compared with histopathologic findings of saturation and targeted prostate biopsies, which served as the reference standard. HIFU: high-intensity focused ultrasound. m: months. SENS: sensitivity. SPEC: specificity. PPV: positive predictive value. NPV: negative predictive value. ACC: accuracy. AUC: area under the curve. 95% CI: 95% confidence interval.

	*Diagnostic performance of vaPSA-D*						
Follow-up interval post-HIFU (n)	**Optimal Cutoff (per Youden’s Index)** **(95% CI)**	**SENS (95% CI)**	**SPEC (95% CI)**	**PPV (95% CI)**	**NPV (95% CI)**	**ACC (95% CI)**	**AUC (95% CI)**
6 m (n = 99)							
	≥ 0.11 (0.08, 0.54)	0.71 (0.29, 1.00)	0.59 (0.49, 0.70)	0.12 (0.06, 0.17)	0.96 (0.92, 1.00)	0.60 (0.51, 0.70)	0.64 (0.39, 0.86)
12 m (n = 74)							
	≥ 0.13 (0.13, 0.18)	0.87 (0.67, 1.00)	0.73 (0.61, 0.85)	0.45 (0.34, 0.59)	0.96 (0.90, 1.00)	0.76 (0.65, 0.85)	0.82 (0.67, 0.94)
36 m (n = 52							
	≥ 0.15 (0.15, 0.24)	1.00 (1.00, 1.00)	0.69 (0.56, 0.82)	0.33 (0.26, 0.47)	1.00 (1.00, 1.00)	0.73 (0.62, 0.85)	0.84 (0.73, 0.93)

**Fig. 1:**
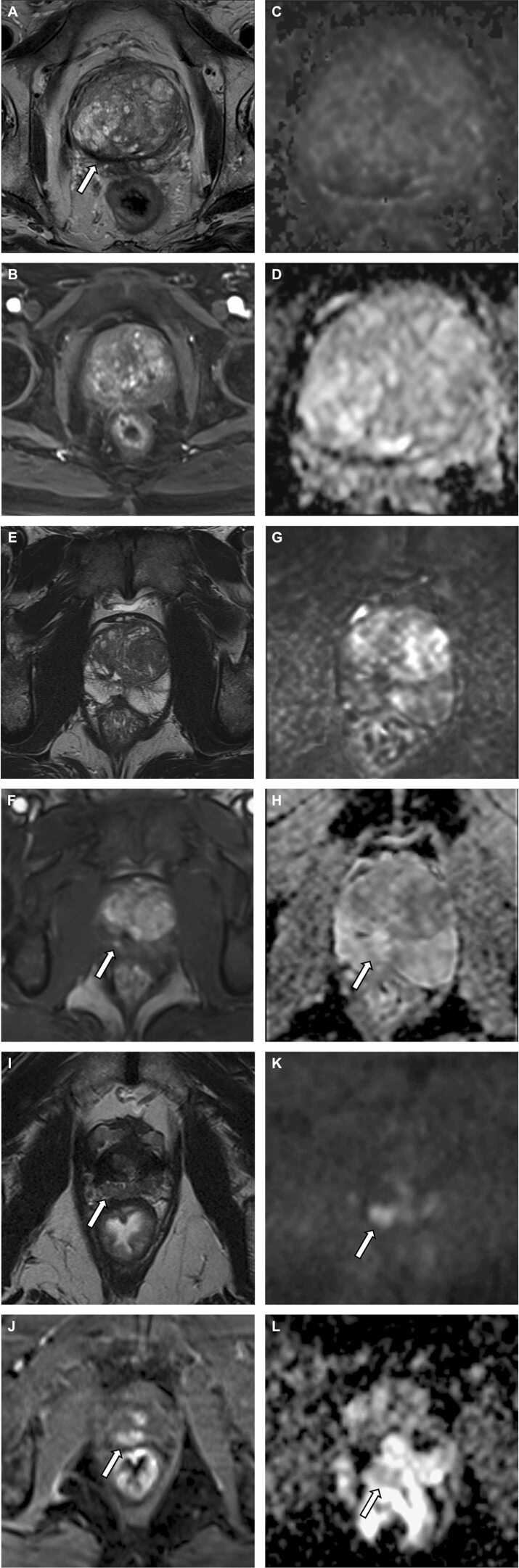
**— Upper panel:** Follow-up mpMRI performed 12 months after HIFU therapy for prostate cancer in the right lateral peripheral zone at the midglandular level. Imaging findings show T2w hypointense residual fibrosis (arrow in A) with no corresponding diffusion restriction (C/D) or enhancement (B), classified as PI-FAB 1. vaPSA-D was 0.10 mg/dl. Subsequent biopsy in combination with histopathology was negative for recurrent prostate cancer. **Middle panel:** Follow-up mpMRI performed 6 months after HIFU therapy for prostate cancer in the right posterior peripheral zone at the apical level. Imaging findings show T2w hypointense residual fibrosis (E) with early, focal, nodular enhancement < 3 mm (arrow in F) that is mildly hypointense on the ADC map (arrow in H) with no corresponding hyperintensity on the high-b-value sequence (G), classified as PI-FAB 2. vaPSA-D was 0.06 mg/dl. Subsequent biopsy in combination with histopathology was negative for recurrent prostate cancer. **Lower panel:** Follow-up mpMRI performed 36 months after HIFU therapy for prostate cancer in the right posterior peripheral zone at the midglandular to apical level. Imaging findings show a focal lesion with low signal intensity on T2w-imaging (arrow in I), early, focal, nodular enhancement (arrow in J), and diffusion restriction (arrows in K/L) within the ablation zone, classified as PI-FAB 3. vaPSA-D was 0.24 mg/dl. Subsequent biopsy in combination with histopathology confirmed in-field recurrence of a clinically significant prostate cancer (ISUP/WHO grade group 2).

**Fig. 2:**
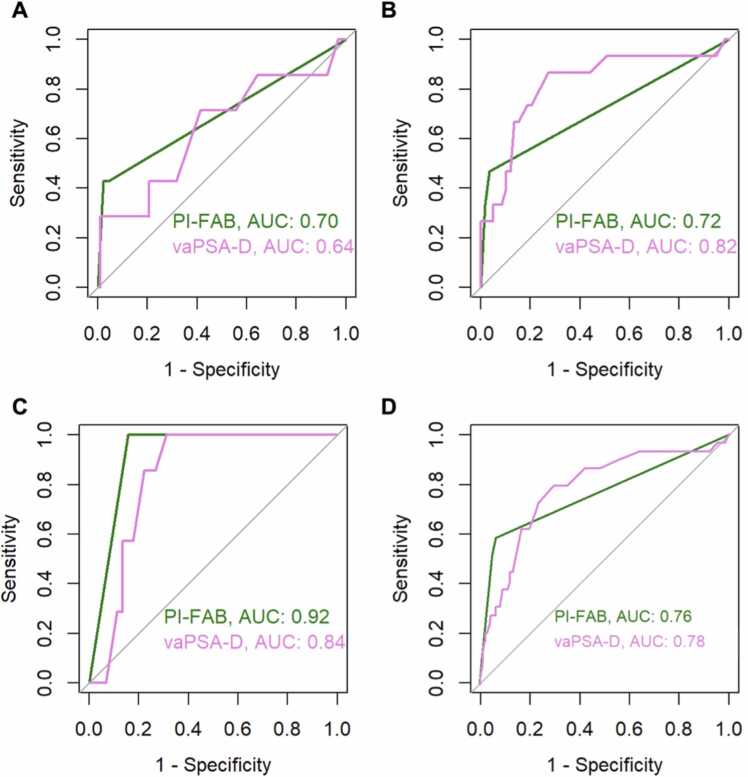
**—** Receiver operating characteristic (ROC) curves showing the overall diagnostic performance of the PI-FAB score (green) and vaPSA-D (pink) at different follow-up intervals (A: 6, B: 12, and C: 36 months) after HIFU treatment, as well as the overall performance (D).

**Fig. 3:**
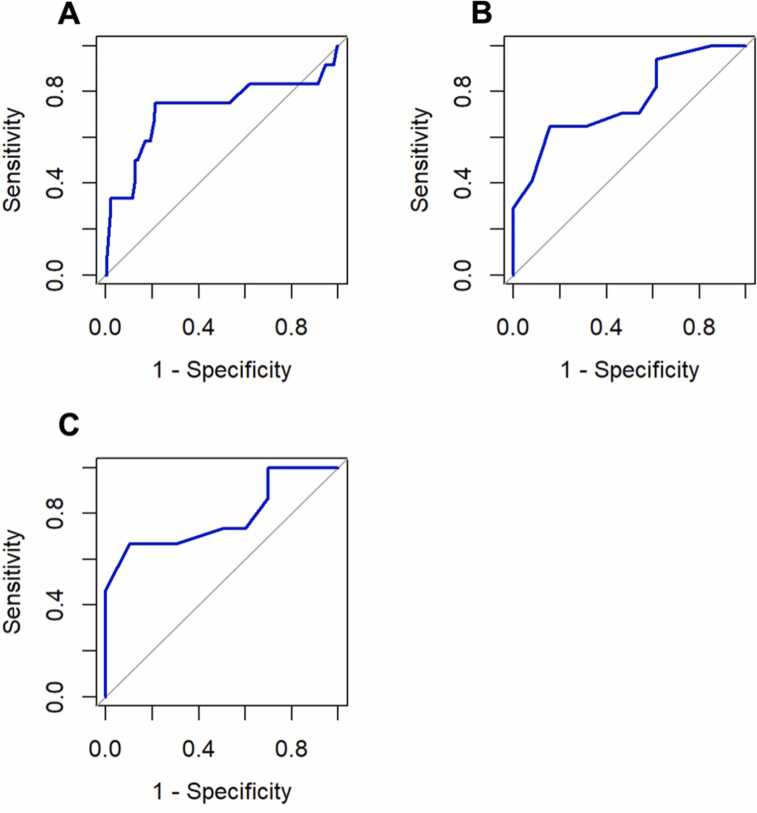
**—** Receiver operating characteristic (ROC) curves illustrating the diagnostic performance of the combined PI-FAB score and vaPSA-D in assessing residual or recurrent prostate cancer after HIFU therapy. The curves correspond to different PI-FAB thresholds: PI-FAB 1, AUC 0.72 (A), PI-FAB ≥ 2, AUC 0.76 (B), PI-FAB 3, AUC 0.78 (C).

When adjusting for multiple observations in the same patient, an overall sensitivity of 72% and specificity of 75% was found with steadily increasing AUC values of 0.64, 0.82, 0.84 at 6, 12 and 36 months after HIFU (overall AUC 0.78), demonstrating an improved diagnostic performance compared to the PI-FAB score at 12 months post-HIFU in our sample. However, the PI-FAB score performed slightly better at 6 and 36 months post-treatment (see ROC curves in Fig. 1, Fig. 2, Fig. 3).

#### 3.1.3 Added value of the combination of PI-FAB and vaPSA-D

In the patient subgroup with a PI-FAB score of 1 (“no suspicion of recurrence”), incorporating vaPSA-D yielded an optimal predicted probability threshold of 0.14 ng/ml for identifying in-field recurrence. At this cut-off, the model achieved a sensitivity of 75% and specificity of 79% (PPV: 19%, NPV: 98%).

In the subgroup of PI-FAB ≥ 2 (“intermediate suspicion of recurrence”), including vaPSA-D resulted in a threshold of 0.18 ng/ml, yielding a sensitivity of 65% and specificity of 85% (PPV: 85%, NPV: 65%).

In the subgroup of PI-FAB score = 3 (“high suspicion of recurrence”), including vaPSA-D resulted in a threshold of 0.18 ng/ml, with an estimated sensitivity of 67% and specificity of 90% (PPV: 91%, NPV: 64%) (see Table 4 for full performance metrics).

**Table 4:** Statistical metrics evaluating the diagnostic performance of the combination of the PI-FAB score and the MRI-based vaPSA-D. SENS: sensitivity. SPEC: specificity. PPV: positive predictive value. NPV: negative predictive value. ACC: accuracy. AUC: area under the curve. 95% CI: 95% confidence interval.

	*Diagnostic performance of the combination of PI-FAB and vaPSA-D*						
PI-FAB	**vaPSA-D threshold (per Youden’s Index)** **(95% CI)**	**SENS (95% CI)**	**SPEC (95% CI)**	**PPV (95% CI)**	**NPV** **(95% CI)**	**ACC (95% CI)**	**AUC** **(95% CI)**
1	**≥ 0.14 (0.14, 0.41)**						
		0.75 (0.50, 1.00)	0.79 (0.72, 0.84)	0.19 (0.12, 0.26)	0.98 (0.96, 1.00)	0.78 (0.72, 0.84)	0.72 (0.51, 0.90)
≥ 2	**≥ 0.18 (0.11, 0.27)**						
		0.65 (0.41, 0.88)	0.85 (0.62, 1.00)	0.85 (0.67, 1.00)	0.65 (0.50, 0.82)	0.73 (0.57, 0.87)	0.76 (0.58, 0.90)
3	**≥ 0.18 (0.11, 0.24)**						
		0.67 (0.40, 0.87)	0.90 (0.70, 1.00)	0.91 (0.75, 1.00)	0.64 (0.50, 0.83)	0.76 (0.60, 0.92)	0.78 (0.58, 0.94)

To quantify the added value of vaPSA-D and account for the correlation due to repeated longitudinal measurements across patients, GEE models were used. Model comparison based on the QIC resulted in a value of 135 for PI-FAB ≥ 2 alone and a substantially lower QIC of 127 when including vaPSA-D, confirming an improved model fit.

## 4 Discussion

Conventional PSA monitoring after HIFU therapy is challenging due to variable residual viable prostate tissue, inflammation, and structural changes [5]. This study evaluated the added value of volume-adjusted PSA density (vaPSA-D), derived from residual vital prostate tissue, in combination with the validated PI-FAB scoring system for detecting in-field recurrence [6], [7], [10].

The PI-FAB score demonstrated an overall good diagnostic performance, particularly at 36 months post-treatment (AUC 0.92), likely due to the progressive resolution of post-treatment changes such as hematoma, edema or contrast enhancement of the ablated area, all of which can complicate post-treatment MRI interpretation and contribute to interpretation variability and errors [21], [22].

vaPSA-D provided consistently high sensitivity and solid, steadily increasing diagnostic accuracy over time (AUC 0.64, 0.82, 0.84 at 6, 12 and 36 months after HIFU, overall 0.78), demonstrating superior diagnostic performance than the PI-FAB score at 12 months post-HIFU. The diagnostic improvement over time likely reflects the evolving structural changes and associated PSA fluctuations induced by HIFU therapy, with high PSA values immediately after treatment caused by edema, necrosis or intraoperative manipulation and scarring becoming more predominant at later time points [14], [21], [23]. The excellent diagnostic ability of vaPSA-D in excluding local recurrence may help to avoid unnecessary biopsies.

For patients classified as intermediate or high risk (PI-FAB ≥ 2), in accordance with the original study, a biopsy may be considered but is not mandatory [6]. In this setting, vaPSA-D provides additional guidance: In cases where vaPSA-D was ≥ 0.18 ng/ml, cancer recurrence was detected with a sensitivity of 65%, suggesting that an elevated vaPSA-D in this setting could serve as a warning sign for recurrence, warranting a biopsy. Conversely, patients with PI-FAB ≥ 2 and a vaPSA-D below this threshold demonstrated a substantially lower recurrence probability, supporting a more conservative management approach that could help avoid unnecessary biopsies without compromising cancer detection. While these thresholds require validation in a larger dataset, they could allow for improved exclusion of recurrence and thus reduce the number of unnecessary biopsies.

Patients classified as high risk (PI-FAB = 3) are commonly recommended for biopsy [6]. This decision can be made with even greater confidence when vaPSA-D is ≥ 0.18, given the high positive predictive value (PPV 0.91).

This study is subject to several limitations. Due to the retrospective, single-center design, the number of patients with complete follow-up decreased over time, primarily due to reluctance to undergo repeated biopsies, which may introduce selection bias. Furthermore, the relatively low prevalence of in-field recurrence within our cohort has affected PPV and NPV, and further subgroup results for these performance measures. As a result, external validation with a larger, independent cohort is essential to confirm the clinical utility of vaPSA-D in routine practice. Due to the small sample size of PI-FAB 2 cases (n = 5), we analyzed PI-FAB ≥ 2 collectively rather than isolating PI-FAB = 2. This may limit the ability to draw specific conclusions regarding intermediate-risk patients. Furthermore, a single reader performed manual slice-per-slice segmentation of the residual viable prostate tissue on mpMRI to calculate vaPSA-D, as this process is quite labor-intensive. However, literature shows excellent inter-reader reproducibility for prostate volume calculation using manual segmentation [24]. Future technical advancements may enable a (semi-)automated approach, reducing workload and improving efficiency. Similarly, PI-FAB scoring was conducted by a single reader; however, prior studies have demonstrated excellent inter-reader agreement for the PI-FAB classification [8], [10].

This study demonstrated that both PI-FAB and vaPSA-D perform well in the post-HIFU setting, each with distinct strengths: PI-FAB shows excellent performance at late follow-up, while vaPSA-D provides high sensitivity throughout all time points, potentially helping to reduce the number of unnecessary biopsies. Most importantly, their combined use offers the most accurate assessment of in-field recurrence after HIFU, supporting a complementary rather than exclusive application of these tools in clinical practice.

## CRediT authorship contribution statement

**Clara Elsner:** Writing – review & editing, Writing – original draft, Visualization, Resources, Investigation, Data curation, Conceptualization. **Toni Rabadi:** Writing – review & editing. **Antonia M. Pausch:** Writing – review & editing, Validation, Investigation, Data curation. **Priska Heinz:** Writing – review & editing, Formal analysis, Data curation. **Ashkan Mortezavi:** Investigation, Data curation. **Andreas M. Hötker:** Writing – review & editing, Supervision, Resources, Project administration, Methodology, Conceptualization. **Ulrike Held:** Writing – review & editing, Methodology, Formal analysis. **Daniel Eberli:** Methodology, Data curation. **Niels J. Rupp:** Investigation, Data curation. **Tabea Borde:** Resources, Data curation.

## Statement for studies in humans/animals

All procedures were performed in compliance with relevant laws and institutional guidelines and have been approved by the appropr

## Declaration of Competing Interest

The authors declare that they have no known competing financial interests or personal relationships that could have appeared to influence the work reported in this paper.
