# Supplementary material for: Prostate cancer: Value of the PI-FAB score and volume-adjusted PSA density in recurrence assessment after HIFU
Source: Eur J Radiol Open. 2026 Jun 25;17:100787. doi: 10.1016/j.ejro.2026.100787 (PMC13320275; doi:10.1016/j.ejro.2026.100787)
Supplement: Supplementary file 1 — Supplementary material [file mmc1.docx]

**Supplementary material**

**
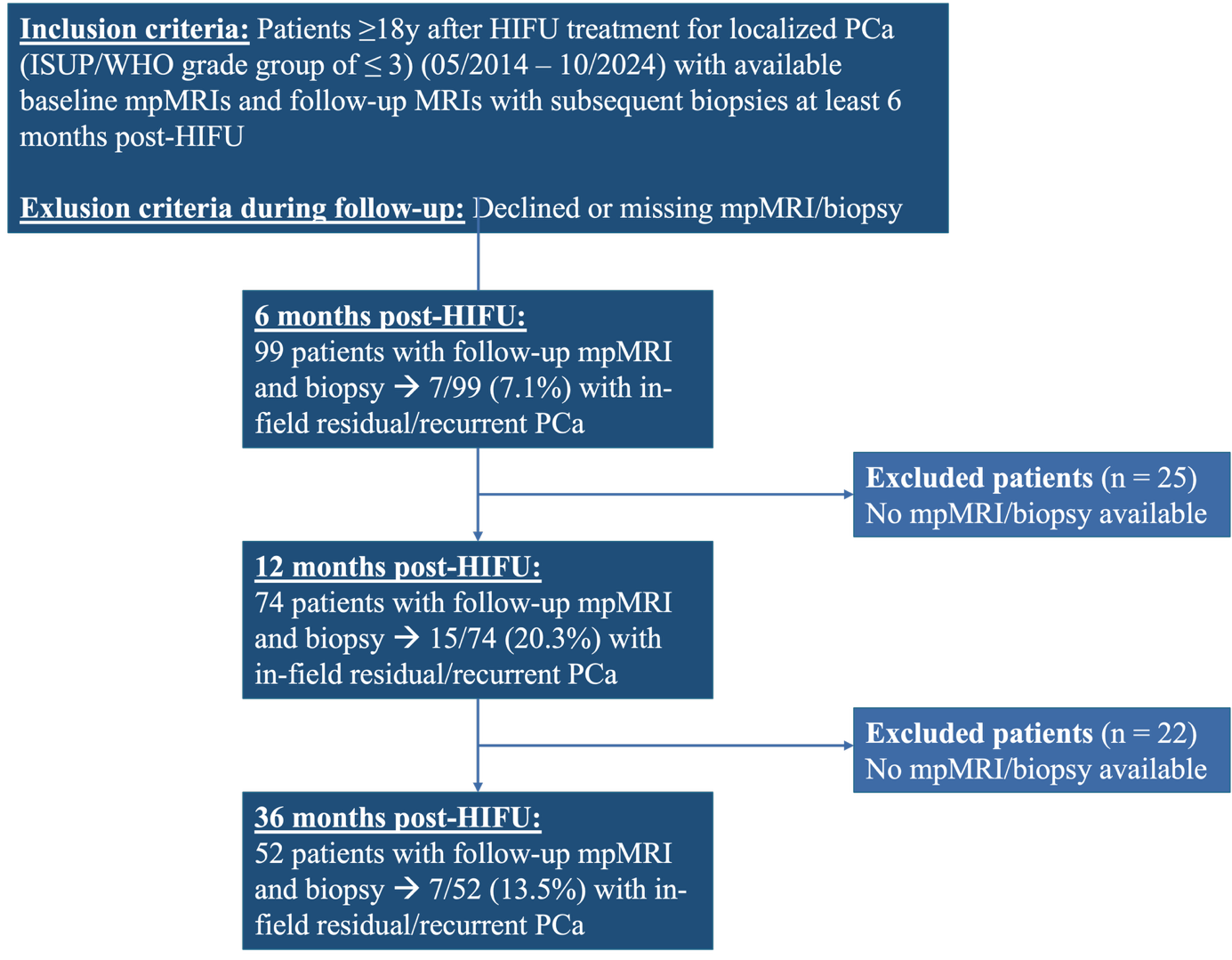
**

**Supplementary Figure S1**: Flowchart of the patient selection process at the different time points of follow-up after HIFU treatment for localized prostate cancer.

| **Follow-up interval post-HIFU** | **Patient cohort size = MRI scans, n** | **Residual/recurrent csPCA, n (%)** | **ISUP/WHO GG** | **n (%)** | **PI-FAB Score** |
| --- | --- | --- | --- | --- | --- |
| **6 m** | 99 | 7/99 (7.1) | 2 | 5 (5.1) | 1 |
|  |  |  |  |  | 1 |
|  |  |  |  |  | 1 |
|  |  |  |  |  | 1 |
|  |  |  |  |  | 3 |
|  |  |  | 4 | 2 (2.0) | 3 |
|  |  |  |  |  | 3 |
| **12 m** | 74 | 15/74 (20.3) | 2 | 10 (13.5) | 1 |
|  |  |  |  |  | 1 |
|  |  |  |  |  | 1 |
|  |  |  |  |  | 1 |
|  |  |  |  |  | 2 |
|  |  |  |  |  | 2 |
|  |  |  |  |  | 3 |
|  |  |  |  |  | 3 |
|  |  |  |  |  | 3 |
|  |  |  |  |  | 3 |
|  |  |  | 3 | 4 (5.4) | 1 |
|  |  |  |  |  | 1 |
|  |  |  |  |  | 1 |
|  |  |  |  |  | 1 |
|  |  |  | 4 | 1 (1.4) | 3 |
| **36 m** | 52 | 7/52 (13.5) | 2 | 5 (9.6) | 3 |
|  |  |  |  |  | 3 |
|  |  |  |  |  | 3 |
|  |  |  |  |  | 3 |
|  |  |  |  |  | 3 |
|  |  |  | 3 | 1 (1.9) | 3 |
|  |  |  | 4 | 1 (1.9) | 3 |

**Supplementary Table S1:** Summary of the patient cohort size and ISUP/WHO grade groups of in-field residual or recurrent prostate cancer along with corresponding PI-FAB scores assigned at the various follow-up intervals after HIFU therapy for localized prostate cancer. The patient count represents the number of MRI scans conducted at each respective follow-up time point.
HIFU: high-intensity focused ultrasound. m: months. csPCa: clinically significant prostate cancer. ISUP/WHO GG: International Society of Urological Pathology/World Health Organization grade group.
